# Brachial-Ankle Pulse Wave Velocity Predicts New-Onset Hypertension and the Modifying Effect of Blood Pressure in a Chinese Community-Based Population

**DOI:** 10.1155/2020/9075636

**Published:** 2020-04-12

**Authors:** Yimeng Jiang, Fangfang Fan, Jia Jia, Jianping Li, Yulong Xia, Jing Zhou, Yong Huo, Yan Zhang

**Affiliations:** Department of Cardiology, Peking University First Hospital, Beijing, China

## Abstract

Brachial-ankle pulse wave velocity (baPWV) was proven to be a prognostic indicator of cardiovascular events. However, the modifying effect of blood pressure (BP) on the longitudinal association between baPWV and new-onset hypertension is not well established. This study included 1849 non-hypertensive Chinese subjects from Shougang cohort study during December 2011 to July 2014. BaPWV was obtained using an Omron Colin BP-203RPE III device. Multivariate logistic regression models were used to evaluate associations of baPWV and outcomes. During a mean follow-up time of 2.36 years, 248 (13.41%) developed hypertension. BaPWV independently and gradably predicted the risk of incident hypertension and the SBP level at revisit (odds ratio or *β* (95% confidence interval) for participants with baPWV in quartile 4 vs. quartile 1: 2.72 (1.54, 4.78) for incident hypertension and 5.92 (4.26, 7.58) for SBP, *P* for trend: <0.001) after adjusting demonstrated risk factors. Besides, the effects of baseline baPWV on either incident hypertension or SBP at revisit were interactively modified by the level of baseline SBP; the effect size increased as the SBP level decreased. baPWV independently predicted the risk of hypertension and BP progression, modified by the level of SBP at baseline in this Chinese community-based population. The combination of baPWV and SBP can help differentiate the potential high-risk candidates who will develop hypertension quickly and benefit from early diagnosis and treatment.

## 1. Introduction

High blood pressure (BP) was the leading risk factor for global disease burden among the 67 risk factors and risk factor clusters in 21 regions worldwide of the Global Burden of Disease study 2010 [1]. In China, hypertension, as the topmost risk factor for cardiovascular disease, especially stroke, is highly prevalent; the prevalence was nearly 45% in the China PEACE Million Persons Project including more than 1.7 million adults from most areas [2] and is still rising due to the unhealthy lifestyle transition. Therefore, the prevention of hypertension at an early stage will be important for the control of CVD and disease burden.

As a maker of artery stiffening, pulse wave velocity (PWV) (either carotid-femoral PWV (cfPWV) or brachial-ankle PWV (baPWV)) can independently predict the risk of CVD morbidity and mortality in different populations [3–6] and is increasingly used in guidelines as a surrogate end point for CVD to investigate asymptomatic target organ damage for hypertensive patients [7, 8]. Actually, PWV was strongly associated with BP levels [9]. However, the causal relation between them is of continuous debate [10]. Early studies demonstrated that elevated BP or poorly controlled hypertension can increase artery stiffness [11, 12], whereas cumulating evidence revealed that higher artery stiffness preceded hypertension and should play an important role in the pathogenesis of arterial hypertension and also the BP-controlling process [13–25].

Previous cohort studies have already elucidated that artery stiffness examined by different techniques like echocardiography [13, 14], cfPWV [15, 19, 20] or baPWV [16–18, 21, 22], and another cardiography device [23] at baseline could predict the progression of BP and the future onset of hypertension in normotensive, untreated, or treated hypertensive individuals, including different kinds (younger or older, health check-up, or community-based) of populations. However, with regard to the effect of BP as a strong predictor of incident hypertension, whether the baseline BP level modifies this relationship warrants further investigation. The present study aimed to longitudinally assess the association of baseline baPWV with new-onset hypertension and the BP level at follow-up, especially the interactive effect of baseline BP level on a Chinese community-based cohort.

## 2. Methods

### 2.1. Study Population

We enrolled participants from the Shougang cohort study proposed to investigate the epidemiology and risk factors of atherosclerosis diseases. This study enrolled community-based participants aged ≥40 years in the Gucheng and Pingguoyuan communities of Shijingshan district in Beijing, China, during December 2011 to July 2014. Detailed study procedures [26] have been reported elsewise. Briefly, 3823 (64.1%) of 5962 participants with genetic testing at baseline responded in the follow-up visit during May 2014 to July 2014. For this analysis, we further excluded participants without baseline baPWV measurements and those who have already been diagnosed as hypertension. Finally, 1839 eligible participants were included in this analysis.

This study was approved by the ethics committee of both Peking University and Peking University First Hospital, and each participant provided written informed consent.

### 2.2. Data Collection

#### 2.2.1. Questionnaire and Anthropometry

All participants were interviewed by trained study coordinators. Standardized questionnaires were used including demographic characteristic, socioeconomic status, health behavior, and medical history. Current smoking was defined as smoking one cigarette per day for no less than half a year. Current drinking was defined as drinking once per week for no less than half a year.

Anthropometric measurements such as height and weight were taken according to standard operating procedures. Body mass index (BMI) was calculated as weight (kilograms) divided by height (meters) squared.

#### 2.2.2. BP Measurements

After a 5-minute rest, seated brachial BP of each participant was accessed using an Omron HEM-7117 electronic sphygmomanometer with standard calibrations. Triplicate consecutive measurements were acquired with ≥1 minute interval between each other. Peripheral systolic BP (SBP) and diastolic BP (DBP) were estimated using these three successive readings' average value.

#### 2.2.3. baPWV Measurements

The baPWV measurement was performed using an oscillometry-based device (BP-203RPE III; Omron Colin, Co., Ltd., Tokyo, Japan) by a trained technician according to a standard protocol as previously described [27]. The details of the oscillometric method have been reported and validated before [28]. Briefly, each participant rested for at least 5 minutes and then lay in the supine position with four occlusion and monitoring cuffs wrapped around both ankles and upper arms. BaPWV, BP, electrocardiogram, and heart sounds were recorded simultaneously. After obtaining bilateral baPWV, the higher one of them was recorded for analysis.

#### 2.2.4. Laboratory Tests

After 12–15 hours of fasting, a venous blood sample was obtained from each participant. Serum or plasma samples were separated within 30 minutes of collection and were stored at −80°C. The Roche C8000 Automatic Analyzer was used for measurement of fasting blood glucose (FBG), the standard 75 g oral glucose tolerance test (OGTT), lipids profiles (including total cholesterol, low-density lipoprotein (LDL) cholesterol, high-density lipoprotein (HDL) cholesterol, and triglycerides), and serum creatinine (Scr) concentrations.

Scr (*μ*mol/L) was measured using an enzymatic method, and then eGFR was estimated using the equation derived from the Chronic Kidney Disease Epidemiology Collaboration (CKD-EPI) [29]: eGFR = 141 × min (Scr/*κ*, 1)^*α*^ × max (Scr/*κ*, 1)^−1.209^ × 0.993^Age^ × 1.018 (if female), where Scr is serum creatinine (mg/dL), *κ* is 0.7 for females and 0.9 for males, *α* is –0.329 for females and –0.411 for males, min indicates the minimum of Scr/*k* or 1, and max indicates the maximum of Scr/*k* or 1.

#### 2.2.5. Definition of Diseases

Hypertension was defined as any self-reported history of hypertension, SBP ≥140 mmHg, DBP ≥90 mmHg, or receiving antihypertensive medications. Diabetes mellitus was defined as any self-reported history of diabetes, FBG ≥7.0 mmol/L, OGTT ≥11.1 mmol/L, or receiving hypoglycemic drugs. Dyslipidemia was defined as any self-reported history of hyperlipidemia, triglyceride (TG) ≥1.70 mmol/L (150 mg/dL), total cholesterol (TC) ≥5.18 mmol/L (200 mg/dL), low-density lipoprotein cholesterol (LDL-C) >3.37 mmol/L (130 mg/dL), high-density lipoprotein cholesterol (HDL-C) <1.04 mmol/L (40 mg/dL), or receiving lipid-lowering medications. CVD was defined as any self-reported history of coronary heart disease, stroke, or transient ischemic attack.

### 2.3. Statistical Analysis

Quantitative data were expressed as mean (standard deviation, SD) for normally distributed variables and median (interquartile range, IQR) for skewed distributed ones. One-way ANOVA was applied to compare normally distributed continuous variables, whereas the Kruskal–Wallis test for skewed distributed ones. Categorical variables were percentages (%) and compared using the Pearson *χ*^2^ test.

Generalized linear regression models were used to investigate the effects of baseline baPWV on the risk of incident hypertension and SBP measured at follow-up. Odds ratios (OR) of incident hypertension and *β* (95% confidence interval, CI) were reported according to 1 m/s increase and quartiles of baPWV levels.

We also performed subgroup and interactive analysis to explore the modification effect of covariates, especially baseline SBP levels, on the relationships between baseline baPWV and BP progression and new-onset hypertension. baPWV ≥1400 m/s was adopted as the cut-off value of increased artery stiffness or arteriosclerosis as previously reported [22, 30].

A *p* value of <0.05 (two-sided) was considered statistically significant for all tests. All analyses were performed using Empower (R) (http://www.empowerstats.com; *X* & *Y* solutions, Inc. Boston, MA) and *R* (http://www.R-project.org).

## 3. Results

### 3.1. Baseline Characteristics

Baseline characteristics of all eligible participants are shown in Table 1 overall and according to categories of baseline SBP with regard to the classification of BP in hypertension guidelines (optimal: <120 mmHg, normal: ≥120 − <130 mmHg, and high normal: ≥130 mmHg). Participants were 54.18 ± 7.54 years old. Of all participants, 31.48% were males. The mean (SD) of BMI was 25.28 ± 3.24 kg/m^2^. The prevalence of diabetes mellitus, dyslipidemia, and CVD were 15.09%, 66.52%, and 5.57%, respectively. Mean (SD) baseline SBP and baPWV were 122.98 ± 9.76 mmHg and 1498.20 ± 276.69 cm/s. Among all participants, 663 (35.86%), 676 (36.56%), and 510 (27.58%) had optimal, normal, and high normal SBP levels, respectively. Participants with high SBP were more likely to be older and males; had higher BMI, DBP, baPWV, TG, FBG, and 2h-PG in OGTT levels, whereas lower HDL-cholesterol level; and had worse kidney function and higher prevalence of current smoking, current drinking, and diabetes mellitus.

### 3.2. Effect of Baseline baPWV on the Risk of New-Onset Hypertension and SBP Measured at Follow-Up

After a mean follow-up time of 2.36 years (median (IQR): 2.40 (2.30–2.40)), 248 (13.41%) developed incident hypertension. Of these, 21 (4.59%), 45 (9.74%), 72 (15.45%), and 110 (23.76%) had incident hypertension among participants with different baseline baPWV levels from quartile 1 (Q1) to quartile 4 (Q4); the risk of incident hypertension was higher among participants with baPWV in the higher quartiles than among those with levels in the lower quartiles (*P* < 0.001).

In Table 2, univariate and multivariate logistic regressions were used to estimate the effect of baseline baPWV on new-onset hypertension. The risk of new-onset hypertension was positively and linearly associated with baseline baPWV level, and this relationship remained statistically significant after adjusting for age, sex, and other covariates including baseline SBP; the OR (95% CI) was 1.14 (1.07–1.20) per 1 m/s increase of baseline baPWV. The risk of new-onset hypertension was also graded related to baseline baPWV as quartiles in multivariate regression model. Compared to participants with the lowest baPWV, higher risks of incident hypertension were observed among those with higher baPWV (OR and 95% CI for Q2 vs. Q1: 1.67 (0.95, 2.94); Q3 vs. Q1: 2.05 (1.18, 3.55); Q4 vs. Q1: 2.72 (1.54, 4.78); *P* for trend: <0.001).

The results of linear regressions for the effect of baseline baPWV on SBP measured at follow-up among 1771 participants without antihypertensive treatment in 2014 were similar as mentioned above (Table 3). SBP at follow-up linearly elevated as the baseline baPWV increased; participants with baPWV in the highest fourth quartile had a SBP level of 5.92 mmHg higher than those with baPWV in the lowest quartile (95% CI: 4.26, 7.58; *P* for trend: <0.001).

### 3.3. Interactive Effect between Baseline SBP and baPWV on the Risk of Incident Hypertension or the SBP Level at Follow-Up

Subgroup and interactive analyses between baseline SBP and baPWV were shown in Table 4. The effects of baseline baPWV on endpoints, either incident hypertension or SBP measured at follow-up, were modified by the level of baseline SBP; the effect size increased as the SBP level decreased. For 1 m/s increase of baPWV, the most rapid increase for the risk of BP progression was found among participants with SBP <120 mmHg (OR and 95% CI: 1.31 (1.15, 1.50) for incident hypertension; *β* and 95% CI: 1.25 (0.86, 1.63) for SBP at follow-up).

No other significant heterogeneity was found among nearly all analyzed subgroups except for lipid-lowering drugs ([Supplementary-material supplementary-material-1]).

### 3.4. Sensitivity Analyses

Logistic regression models, but not Cox models, were used because all participants were recruited and revisited centrally within ∼3 months; the follow-up duration was much the same. Nevertheless, we further did multivariate regressions adjusting the follow-up time (data not shown), and the main results remained statistically significant.

## 4. Discussion

In this study, we longitudinally examined the association of artery stiffness with future new-onset hypertension and the progression of BP in a Chinese community-based cohort. Two main findings were reached as follows: first, the incidence of hypertension and the SBP level at revisit gradually increased with the elevation of baseline baPWV, and this relation was independent of other traditional risk factors for CVD including baseline SBP. Second, interactive effect was found between baPWV and SBP on the risk of SBP progression and incident hypertension, that is to say, a more predominant effect on individuals with optimal SBP levels.

Our finding of the relation between baPWV, as an indicator of artery stiffness, and an increased risk of hypertension or BP progression is consistent with previous studies [13–23]. Early in ARIC study [13], data of 6 years' follow-up among 6992 normotensive individuals aged 45–64 years revealed that arterial elasticity, measured from B-mode ultrasound of carotid artery, was associated with 15% elevated risk of hypertension per 1 SD decrease. As the “gold standard” method for central artery stiffness, cfPWV at baseline was independently related to the longitudinal increase of SBP or incident hypertension in 449 BLSA (the Baltimore Longitudinal Study of Aging) participants for a 4.9-year follow-up, in 1759 community-based population of Framingham Heart Study over a 7-year period, etc. [15, 19, 20]. Similarly, baPWV [16–18, 21, 22], an alternatively developed method representing a combination stiffness of elastic and muscular arteries, was also proven to be a precursor in several longitudinal cohort studies mainly among East Asians. Recently, the PWV, measured by a whole-body impedance cardiography device, and BP progression association was also proven robust in 1449 young Finnish adults aged 30–45 years [23]. Therefore, the recommendations from the American Heart Association inclined to believe that arterial stiffness represents an antecedent factor rather than a consequence of hypertension in consideration of strong evidence from several related studies [31].

The mechanism for the artery stiffness and longitudinal BP progression is not completely clear and warrants further researches. Nevertheless, several plausible mechanisms may explain this relationship. The main pathophysiology of a stiffened vasculature is the disrupting elastin in the artery wall, which was proven to predate the subsequent development of hypertension both in mouse models [31] and patients [32] with elastin haploinsufficiency. Besides, high artery stiffness is associated with impaired baroreceptor sensitivity and increased BP lability [33, 34], resulting in potentially marked alterations of BP accompanying the change of cardiac output during daily living [32–35].

Adding to prior reports on the existence of association between baPWV and BP increment or incident hypertension, our study is novel in showing that baseline SBP could modify this interrelationship. It is worth noting that there was obvious interactive effect between baPWV and SBP, and a stronger predicting value of baPWV existed in individuals with optimal SBP level, in accordance with the data in normotensive participants of a Korean genome and epidemiology study [22]. One possible speculation is that in those with optimal SBP level, baPWV as a representor of other risk factor clusters may have more important role than in those with higher SBP levels; however, we have already adjusted other common covariates, and the baseline characteristics of the optimal SBP group were related to a low arteriosclerosis risk. Therefore, increased baPWV may reflect other factors that were not included in this study, and the underlying mechanism for this discrepancy should be further verified and investigated.

Actually, cfPWV is the most recognized and established index and considered as the “gold standard” for aortic stiffness assessment. However, its measurement requires a high level of skill and exposure of the inguinal region, which largely limit its applicability in clinical practice, especially in a large population research. BaPWV used in our study is an oscillometric technique for measuring arterial stiffness in large- and medium-sized arteries, which is automated and noninvasive and can be applied easily in general population studies. Previous studies have shown that baPWV showed a close correlation with cfPWV [28]. What is more, as an indicator both for large-sized central elastic arterial and medium-sized peripheral muscular arterial stiffness, baPWV may be more representative than cfPWV of arterial load [36]. Until now, a number of studies have revealed that baPWV was also an independent predictor for cardiovascular diseases. An increase in baPWV by 1 m/s corresponded with an increase of 12%, 13%, and 6% in total cardiovascular events, cardiovascular mortality, and all-cause mortality in meta-analysis of 18 studies including 8169 participants [5].

Several limitations to our study should be acknowledged. First, this observational study does not provide a causal relation between baPWV and the development of hypertension, and this interrelationship should be further explored in intervention study to investigate the change of incident risk of hypertension by lowering artery stiffness. Second, this analysis is based on a short follow-up duration that was no more than 3 years. Consequently, whether the main results were also applied to a long-term examination will be doubted. Third, incident hypertension and BP change were identified by only two surveys (the baseline and the 2014 follow-up examination), so detailed BP information such as BP variations cannot be obtained in our analysis. Finally, our Chinese community-based population made the ability to generalize these results to other race-ethnic populations limited.

In summary, our study has demonstrated that artery stiffness evaluated by baPWV independently and gradably predicted the risk of incident hypertension and the SBP level at revisit in this Chinese community-based population. Furthermore, the baseline value of SBP can modify the predicting effect of baPWV on the risk of incident hypertension and SBP progression. Consequently, the combination of the levels of baPWV and SBP can help differentiate the potential high-risk candidates who will develop new-onset hypertension quickly and benefit from early diagnosis and treatment. With regard to the large burden of hypertension worldwide, our study reinforces the importance of regular screening of baPWV in community-based populations and making effort to develop strategies to prevent new-onset hypertension as early as possible.

## Figures and Tables

**Table 1:** Baseline characteristics of all eligible participants.

Variables	Total (*n* = 1849)	Baseline SBP, mmHg			*P* value
		<120 (*n* = 663)	≥120 − <130 (*n* = 676)	≥130 (*n* = 510)	
Age, year	54.18 ± 7.54	52.60 ± 6.79	54.25 ± 7.23	56.15 ± 8.36	<0.001
Males, *n* (%)	582 (31.48%)	149 (22.47%)	243 (35.95%)	190 (37.25%)	<0.001
BMI, kg/m^2^	25.28 ± 3.24	24.53 ± 3.11	25.56 ± 3.27	25.87 ± 3.19	<0.001
SBP, mmHg	122.98 ± 9.76	112.43 ± 6.06	124.70 ± 2.87	134.40 ± 2.81	<0.001
DBP, mmHg	71.57 ± 7.61	67.16 ± 6.28	73.04 ± 6.55	75.33 ± 7.73	<0.001
baPWV, cm/s	1498.20 ± 276.69	1381.52 ± 221.81	1508.78 ± 263.42	1635.88 ± 291.68	<0.001
Total cholesterol, mmol/L	5.34 ± 0.98	5.31 ± 0.95	5.34 ± 0.99	5.38 ± 1.01	0.449
Triglyceride^*∗*^, mmol/L	1.19 (0.86–1.78)	1.11 (0.82–1.60)	1.24 (0.87–1.84)	1.31 (0.91–1.82)	<0.001
HDL-cholesterol, mmol/L	1.48 ± 0.39	1.54 ± 0.40	1.46 ± 0.38	1.43 ± 0.39	<0.001
LDL-cholesterol, mmol/L	3.25 ± 0.82	3.20 ± 0.80	3.28 ± 0.82	3.30 ± 0.86	0.083
Fasting glucose, mmol/L	5.87 ± 1.52	5.71 ± 1.61	5.87 ± 1.40	6.09 ± 1.53	<0.001
2 h-PG in OGTT, mmol/L	7.75 ± 3.51	7.32 ± 3.37	7.65 ± 3.37	8.43 ± 3.77	<0.001
eGFR, ml/min/1.73 m^2^	97.33 ± 11.21	99.00 ± 10.42	96.80 ± 11.59	95.87 ± 11.42	<0.001
Current smoking, *n* (%)	371 (20.06%)	105 (15.84%)	156 (23.08%)	110 (21.57%)	0.003
Current drinking, *n* (%)	424 (22.93%)	130 (19.61%)	171 (25.30%)	123 (24.12%)	0.035
Prevalence of disease, *n* (%)					
Diabetes mellitus	279 (15.09%)	72 (10.86%)	105 (15.53%)	102 (20.00%)	<0.001
Dyslipidemia	1230 (66.52%)	424 (63.95%)	461 (68.20%)	345 (67.65%)	0.212
Cardiovascular disease	103 (5.57%)	31 (4.68%)	34 (5.03%)	38 (7.45%)	0.090
Medication, *n* (%)					
Hypoglycemic agents	112 (6.06%)	36 (5.44%)	38 (5.63%)	38 (7.45%)	0.301
Lipid-lowering agents	97 (5.28%)	31 (4.70%)	36 (5.36%)	30 (5.94%)	0.642

baPWV, brachial-ankle pulse wave velocity; BMI, body mass index; SBP, systolic blood pressure; DBP, diastolic blood pressure; eGFR, estimated glomerular filtration rate; HDL, high-density lipoprotein; LDL, low-density lipoprotein; 2 h-PG in OGTT: 2-hour postload glucose in oral glucose tolerance test. ^*∗*^Median (interquartile range).

**Table 2:** Multivariate regression for the effect of baseline baPWV on the development of incident hypertension.

Variables	*N*	Incidence, %	Crude model		Adjusted model 1		Adjusted model 2	
			OR (95% CI)	*P* value	OR (95% CI)	*P* value	OR (95% CI)	*P* value
baPWV continuous, per 1 m/s increase								
	1849	248 (13.41%)	1.23 (1.17, 1.28)	<0.001	1.22 (1.15, 1.28)	<0.001	1.14 (1.07, 1.20)	<0.001

baPWV categories, cm/s								
Q1 (<1311)	466	21 (4.59%)	1 (reference)		1 (reference)		1 (reference)	
Q2 (≥1311 − <1451)	462	45 (9.74%)	2.25 (1.32, 3.83)	0.003	2.15 (1.26, 3.68)	0.005	1.67 (0.95, 2.94)	0.076
Q3 (≥1451 − <1622)	466	72 (15.45%)	3.80 (2.30, 6.30)	<0.001	3.50 (2.09, 5.87)	<0.001	2.05 (1.18, 3.55)	0.010
Q4 (≥1622)	463	110 (23.76%)	6.48 (3.98, 10.56)	<0.001	5.49 (3.24, 9.28)	<0.001	2.72 (1.54, 4.78)	<0.001
*P* for trend				<0.001		<0.001		

baPWV, brachial-ankle pulse wave velocity; OR, odds ratio; CI, confidence interval. Model 1, adjusted for age and sex; Model 2, adjusted for variables in model 1 and body mass index, current smoking, current drinking, baseline systolic blood pressure and estimated glomerular filtration rate, diabetes mellitus, dyslipidemia, history of cardiovascular disease, hypoglycemic agents, and lipid-lowering agents.

**Table 3:** Multivariate regression for the effect of baPWV on SBP measured at revisit in participants without antihypertensive treatment in the follow-up survey.

Variables	*N*	Crude model		Adjusted model 1		Adjusted model 2	
		*β* (95% CI)	*P* value	*β* (95% CI)	*P* value	*β* (95% CI)	*P* value
baPWV continuous, per 1 m/s increase							
	1771	1.97 (1.77, 2.17)	<0.001	0.94 (0.72, 1.17)	<0.001	0.98 (0.75, 1.20)	<0.001

baPWV categories							
Q1 (<1311)	443	1 (reference)		1 (reference)		1 (reference)	
Q2 (≥1311 − <1451)	438	4.75 (3.19, 6.31)	<0.001	1.64 (0.24, 3.04)	0.022	1.48 (0.08, 2.88)	0.039
Q3 (≥1451 − <1622)	447	8.78 (7.22, 10.33)	<0.001	3.06 (1.59, 4.53)	<0.001	2.95 (1.48, 4.42)	<0.001
Q4 (≥1622)	443	13.97 (12.41, 15.53)	<0.001	5.75 (4.1, 7.39)	<0.001	5.92 (4.26, 7.58)	<0.001
*P* for trend		<0.001		<0.001		<0.001	

baPWV, brachial-ankle pulse wave velocity; SBP, systolic blood pressure; OR, odds ratio; CI, confidence interval. Model 1, adjusted for age, sex, and baseline SBP; Model 2, adjusted for variables in model 1 and body mass index, current smoking, current drinking, baseline estimated glomerular filtration rate, diabetes mellitus, dyslipidemia, history of cardiovascular disease, hypoglycemic agents, and lipid-lowering agents.

**Table 4:** Interactive effect between baseline SBP and baPWV on the risk of incident hypertension or the SBP level at follow-up.

Variables	*N*	Incident hypertension			*P* value^*∗*^	SBP measured at follow-up			
		Incidence, %	OR (95% CI)	*P* value		*N*	*β* (95% CI)	*P* value	*P* value^*∗*^
Baseline SBP, mmHg									
<120	663	30 (4.52%)	1.31 (1.15, 1.50)	<0.001		652	1.25 (0.86, 1.63)	<0.001	
≥120 − <130	676	76 (11.24%)	1.16 (1.06, 1.26)	<0.001		648	0.96 (0.62, 1.30)	<0.001	
≥130 − <140	510	142 (27.84%)	1.08 (1.00, 1.16)	0.050	0.007	471	0.76 (0.42, 1.10)	<0.001	0.047

baPWV, brachial-ankle pulse wave velocity; SBP, systolic blood pressure; OR, odds ratio; CI, confidence interval. Variables in multivariate adjusted models, age, sex, body mass index, current smoking, current drinking, baseline SBP and estimated glomerular filtration rate, diabetes mellitus, dyslipidemia, history of cardiovascular disease, hypoglycemic agents, and lipid-lowering agents. ^*∗*^*p* value for interaction trend test.

## Data Availability

Full study protocol (approved by the Research Ethics Committee) and patient level data are available from Chief Investigator, Professor Yan Zhang (drzhy1108@163.com).

## Supplementary Materials

Supplementary Materials Table S1: subgroup analyses for the effect of baPWV on the development of incident hypertension (*n* = 1849).
