# Supplementary material for: Brachial-Ankle Pulse Wave Velocity Predicts New-Onset Hypertension and the Modifying Effect of Blood Pressure in a Chinese Community-Based Population
Source: Int J Hypertens. 2020 Apr 12;2020:9075636. doi: 10.1155/2020/9075636 (PMC7174949; doi:10.1155/2020/9075636)
Supplement: Supplementary Materials — Table S1: subgroup analyses for the effect of baPWV on the development of incident hypertension (n = 1849). [file 9075636.f1.doc]

**Supplemental Data**

**Table S2. Subgroup analyses for the effect of baPWV on the development of incident hypertension (n=1849)**

| **Variables** | **N** | **Incidence, %** | **OR (95%CI)^*^** | ***P* for interaction** |
| --- | --- | --- | --- | --- |
| Sex |  |  |  |  |
| Male | 582 | 94 (16.15%) | 1.09 (1.00, 1.18) | 0.160 |
| Female | 1267 | 154 (12.15%) | 1.17 (1.09, 1.25) |  |
| Age, years |  |  |  |  |
| <60 | 1500 | 54 (10.02%) | 1.15 (1.07, 1.25) | 0.505 |
| ≥60 | 349 | 194 (14.81%) | 1.11 (1.03, 1.21) |  |
| BMI, kg/m^2^ |  |  |  |  |
| <28 | 1513 | 176 (11.63%) | 1.13 (1.06, 1.21) | 0.868 |
| ≥28 | 336 | 72 (21.43%) | 1.14 (1.03, 1.27) |  |
| Current smoking |  |  |  |  |
| No | 1478 | 191 (12.92%) | 1.15 (1.08, 1.23) | 0.277 |
| Yes | 371 | 57 (15.36%) | 1.08 (0.97, 1.20) |  |
| Current drinking |  |  |  |  |
| No | 1425 | 180 (12.63%) | 1.15 (1.08, 1.22) | 0.255 |
| Yes | 424 | 68 (16.04%) | 1.07 (0.95, 1.20) |  |
| eGFR, mmol/L |  |  |  |  |
| <90 | 401 | 66 (16.46%) | 1.14 (1.05, 1.23) | 0.923 |
| ≥90 | 1446 | 182 (12.59%) | 1.14 (1.06, 1.23) |  |
| Diabetes mellitus |  |  |  |  |
| No | 1570 | 192 (12.23%) | 1.15 (1.08, 1.22) | 0.498 |
| Yes | 279 | 56 (20.07%) | 1.10 (1.00, 1.22) |  |
| Dyslipidemia |  |  |  |  |
| No | 619 | 61 ( 9.85%) | 1.14 (1.03, 1.25) | 0.984 |
| Yes | 1230 | 187 (15.20%) | 1.14 (1.07, 1.21) |  |
| Cardiovascular disease |  |  |  |  |
| No | 1746 | 225 (12.89%) | 1.14 (1.08, 1.21) | 0.470 |
| Yes | 103 | 23 (22.33%) | 1.08 (0.93, 1.25) |  |
| Hypoglycemic drugs |  |  |  |  |
| No | 1735 | 230 (13.26%) | 1.12 (1.06, 1.19) | 0.057 |
| Yes | 112 | 18 (16.07%) | 1.33 (1.11, 1.58) |  |
| Lipid-lowering drugs |  |  |  |  |
| No | 1739 | 227 (13.05%) | 1.16 (1.10, 1.23) | 0.004 |
| Yes | 97 | 20 (20.62%) | 0.90 (0.75, 1.08) |  |

**Abbreviations:** baPWV, brachial-ankle pulse wave velocity; OR, odds ratio; CI, confidence interval.

**^*^:** OR for incident hypertension per 1 m/s increase of baseline baPWV.

**Variables in the model:** age, sex, body mass index, current smoking, current drinking, baseline systolic blood pressure and estimated glomerular filtration rate, diabetes mellitus, dyslipidemia, history of cardiovascular disease, hypoglycemic agents, and lipid-lowering agents.
